# Persons with rheumatoid arthritis challenge the relevance of the health assessment questionnaire: a qualitative study of patient perception

**DOI:** 10.1186/s12891-017-1566-5

**Published:** 2017-05-12

**Authors:** David Ebbevi, Anna Essén, Helena Hvitfeldt Forsberg

**Affiliations:** 1 Department of Learning, Informatics, Management and Ethics, Karolinska Institute, Tomtebodavägen 18A, 171 77 Stockholm, Sweden; 2 Center for Human Resource Management and Knowledge Work, Stockholm School of Economics, Stockholm, Sweden

**Keywords:** Rheumatoid Arthritis, Thematic analysis, Outcomes assessment, Qualitative Research, Patient reported outcome measures

## Abstract

**Background:**

The Stanford Health Assessment Questionnaire-Disability Index (HAQ) is widely used to measure functional ability in persons with Rheumatoid Arthritis (RA). The instrument was developed with limited involvement from persons with RA, and their perception of the instrument has not been studied in depth. The aim of this study was to explore how persons with RA experience the use of the HAQ in care.

**Methods:**

The study used secondary data analysis. Persons with RA participated in semi-structured interviews in previous research projects. Thirty-nine interviews were included based on data fit, and thematic analysis applied.

**Results:**

The participants questioned the relevance of the HAQ but nevertheless experienced that the instrument had a profound effect on their understanding of health and how care is delivered. The analysis resulted in three themes: *Problems with individual items*, *meaning of the summative score*, and *effects on care and health perceptions*.

**Conclusions:**

To make the HAQ relevant to persons with RA, it needs to be revised or to include an option to select items most meaningful to the respondent. To ensure relevance, the HAQ update should preferably be co-created by researchers, clinicians and persons with RA.

## Background

Health outcome measurement has long been the focus of quality assessment in rheumatology [1]. The Stanford Health Assessment Questionnaire-Disability Index (HAQ) is a commonly used instrument to assess functional ability in Rheumatoid Arthritis (RA). It has been extensively validated [2], recommended by the American College of Rheumatology [3] and measured in the majority of studies of RA treatments [4]. Patients’ perceptions of other instruments used in rheumatology have been qualitatively evaluated in depth [5, 6], but perceptions of the HAQ need further investigation.

During development of the HAQ, patient feedback identified imprecisions and ambiguity in the instrument, but patients did not take part in the *initial* stages of its development [1]. Excluding patients from initial development stages is common [7] but problematic because patient feedback is limited to the items researchers hypothesize as important. In the same manner, patient preferences among the HAQ items have been evaluated quantitatively [8, 9]. These studies mainly illustrated patients’ perceptions of existing items rather than investigating which functional aspects patients would find relevant to measure. Several qualitative studies mentioned limitations of the HAQ from the patient’s perspective [10–13]. However, these studies did not have the specific aim to explore the HAQ and, consequently, did not show how patients experienced the instrument or what consequences the use of the HAQ may generate. Therefore, the aim of this study was to explore how persons with RA experience the use of the HAQ in care.

## Methods

This section will provide a brief description of the HAQ, followed by methodology, design, study participants, data assessment, and analysis.

### Empirical setting: Swedish translation of the HAQ and its use in RA care

The HAQ [14] consists of 20 items grouped into eight categories (Table 1). Each category concerns a functional area represented by two to three items. The items aim to capture specific functional abilities and cover all major joints of the body. For each item, the patient is asked to rate the difficulty on a six-point scale. Each item is assigned a score from 0 to 3: 0 points (“without any difficulty”), 1 point (“with some difficulty”), 2 points (“with much difficulty,” “with a special device” or “with help from another person”), 3 points (“unable to do”) [15]. The HAQ score is calculated as the average of the highest score from each category. Hence, the HAQ score ranges from zero to three with a high score representing substantial difficulties.

**Table 1:** Back-translation of the Swedish HAQ

Category	Are you able to…
Dressing and grooming	shampoo your hair? dress yourself, including tying shoelaces and doing buttons?
Arising	stand up from an armless straight chair? get in and out of bed?
Eating	cut meat?* lift a full cup or glass to your mouth? prepare your own meal?**
Walking	walk outdoors on flat ground? climb down five steps?***
Hygiene	wash and dry your entire body? take a tub bath? get on and off the toilet?
Reach	reach and get down a two-kilogram bag of, for example, sugar from just above your head? bend down to pick up clothing from the floor?
Grip	open car doors? open jars which have been previously opened? turn faucets on and off?
Other activities	run errands and shop? get in and out of a car? vacuum?^a^

*Notes*: Based on translation by Ekdahl et al. [15]. Four items deviate from the original [14] as follows: *“cut your meat?” **“open a new milk carton?” ***“climb up five steps?”

^a^“do chores such as vacuuming or yardwork?”

In Sweden, RA is managed through publicly funded interprofessional specialist care. A majority of patients participate in the Swedish rheumatology quality registry, which stores data on clinical procedures and outcomes, as well as patient-reported outcome measures (e.g., the HAQ). Prior to appointments, patients fill in the HAQ electronically, either from home or in the waiting room. Most patients complete the HAQ once or twice per year. Only one answer per item is possible and at least one item in each category must be answered. Typically, the patient discusses the self-reported HAQ with the physician during the appointment. A graphical interface shows longitudinal trends.

### Methodology and design

A qualitative design was chosen for this study to capture experiences of HAQ use that were as true to patients’ experiences as possible [16]. It has been argued that this design is necessary to assess the patient value of a patient-reported outcome measure [17]. The study was based on 39 semi-structured interviews collected in two earlier studies [18, 19] (i.e., a secondary data analysis [20]). The specific material was chosen because the data contained rich patient accounts of the HAQ. The first and second authors of the present study conducted the interviews. A secondary analysis where the researchers collected the original data has been termed ‘analytic expansion’ [20] or ‘supplementary analysis’ [21]. This design has the advantage that tacit context knowledge can be transferred from the earlier studies to the new analysis [20]. The studies were approved by the Regional Ethics Board in Stockholm (reg.nr: 2009.895-31.5 and 2012.1911-31.5).

### Study participants and data assessment

Participants of the earlier studies had RA and were purposefully sampled to create diversity in age, gender, disease severity, disease duration and satisfaction with care (i.e., maximum variation sampling [16]). The first study [18] concerned patients’ overall experiences of RA, including the structure, process and outcome of RA care. The second study [19] concerned patients’ experiences of the process of care, including the usability and deployment of the electronic system patients use to enter self-reported HAQ data. The first author of the present study assessed ‘data fit’ of interviews (i.e., appropriateness of the data to the research question of the present study) [21]. All interviews with rich accounts on functional aspects relevant to the assessment of HAQ, or perceptions of HAQ usage were included (i.e., purposive sampling [16]). From the first study 21 interviews were included, and from the second 18. Original tape recordings were available from the first study, but not from the second. Five interviews from the second study had been lost and were not assessed for inclusion.

### Analysis

The respective authors transcribed their interviews, and the first author thematically analyzed the data to capture manifest and latent content using the method described by Braun and Clarke [22]. The process is outlined with examples in Table 2, and included inductive coding in NVivo v.10 followed by grouping through constant comparison. The themes were defined to maximize inner homogeneity and outer heterogeneity [16]. Even though described as a linear process, the themes were created iteratively, moving back and forth between the steps outlined in Table 2. Further, as themes rather than categories, they are not necessarily mutually exclusive with respect to a meaning unit (i.e., a meaning unit may represent more than one theme). The first author did the analysis. Member checks and investigator triangulation was not used. When faced with ambiguities, interpretation was facilitated by Watzlawick et al.’s communication theory [23]. For example, participant dissatisfaction followed by expressions such as “I might think, kind of…” was not interpreted as the participants being unsure of their dissatisfaction, but rather as maintaining a relationship with the interviewer. Thus, attention was paid to reflexivity of the interview situation, and in analysis latent meaning influenced the conceptualized themes [24]. Data saturation was achieved in terms of the themes found, but not in terms of the content of the themes; new aspects of the themes arose late in analysis, namely additional examples of perceptions of specific items.

**Table 2:** Thematic analysis

Step	Description	Example from analysis in the present study
Familiarizing	Reading the data multiple times and noting ideas	Ideas: • The instrument affects what is spontaneously seen as functional ability. Is this reflexivity or validity? • Many items seems to be missing from HAQ • HAQ complements other measurements
Coding	Applying open codes to data relevant to the research question	Coding: • “I wish I could dress in what I wanted by myself. Just that is important. I would like to be able to shower myself, wash my hair, these kinds of practical things. Caring for myself, that would be most important” was coded as “wishing to dress, shower and wash hair by oneself”
Searching for themes	Grouping codes into initial subthemes and themes	Forming tentative subthemes (here with example of codes): • *Function affects and is affected by the HAQ* ^a^ o cleaning and cutting meat o wishing to dress, shower and wash hair by oneself o difficulty biking, drinking coffee and wiping the stove • *Missing items* o Questions missing can be discussed at the appointment o HAQ is so narrow, I’m declared cured
Reviewing	Checking if the themes represent their codes and all relevant data	Merging subthemes and moving codes: • The subtheme *Function affects and is affected by the HAQ* ^a^ was incorporated into *Missing items* because the data as a whole did not clearly support reflexivity of items • The code “HAQ is so narrow, I’m declared cured” was moved back and forth between *Static items* and *Missing items* and ended up at *Missing items*
Defining	Analysis for renaming themes and formulating an explicit definition	Renaming: • Subtheme *Effect on behavior* clarified as *Effects on physician behavior* • A theme, *A wish to change the HAQ*, concerned only experiences of the individual items and not the participants’ wishes, so instead was renamed *Problems with individual items*

*Notes*. Inductive analysis [20] in the present study, subthemes in italics. Some data demonstrate more than one theme (e.g., the code “HAQ is so narrow, I’m declared cured”)

^a^This theme concerned an early hypothesis that regular use of the HAQ would affect participants’ perceptions of function, i.e., drawing attention to abilities in HAQ would make respondents more attentive to the abilities even though they were not important prior to exposure to HAQ (reflexivity)

## Results

The data suggested patients had not only heterogeneous experiences of the use of HAQ, but also commonalities. The various experiences salient in the data were grouped into the three themes outlined in Table 3.

**Table 3:** Summary of findings

Themes and subthemes	Defining the experience of…
Problems with individual items	frustration when responding to HAQ items
Missing items	wanting to include items in the HAQ not currently there
Unclear items	not knowing how to interpret a specific item
Unnecessary items	answering items perceived as not meaningful
Static items	the same items being scored every time the instrument is used
Meaning of the summative score	frustration and insights when trying to understand the summative score
Capturing overall function	how the HAQ may capture a complete picture of function
Reflecting a temporary state	the HAQ as a snapshot of function
Requiring strategies for interpretation	understanding the score in relation to other measurements or the physician’s interpretation
Effects on care and health perceptions	positive and negative effects from use of the HAQ in care
Effects on physician behavior	how use of the HAQ would cause some physician behavior
Effects on understanding of RA	how use of the HAQ would cause understanding of health

Many aspects of the themes below are illustrated with quotes and, if contextually relevant, a short description of the participant behind the quote. Parenthesis after quotes show: Woman (W)/Man (M); age in years; and years since RA debut.

### Problems with individual items

The individual items of the HAQ created much frustration for participants, leading them to describe the HAQ by many labels: “old-fashioned,” “childish,” “silly,” “narrow,” “retired-like,” “too general,” “unnecessary” or “dead boring.” Despite positive experiences, negative experiences dominated the data. The results below are therefore presented as subthemes related to experienced negative properties of items: missing, unclear, unnecessary or static.

### Missing items

Participants were asked about activities important to them before being primed by questions about the HAQ. Their responses included factors present in the HAQ: opening jars, cutting meat, using the toilet, showering, taking baths, getting dressed, tying shoelaces, vacuuming and cooking. Responses also included factors not covered by the HAQ: wringing out a soaked washcloth, holding a cup of coffee, dressing a child, opening locked doors, sitting for a long time, brushing hair, filling up the car, doing yardwork (present in original HAQ but not in the Swedish translation, see Table 1), bicycling, writing, painting and kneading dough. Typically, participants experienced limitations of the instrument but had difficulty presenting alternative items:

I cannot come up with some concrete proposal of how it should be instead. Not right now anyway. Erhm, but some issues I think are very coarse.…I would think that the wording could perhaps be changed on some things. (W, 49, 29)

When asked directly about what was missing, respondents often suggested measuring phenomena that are not functional, such as tiredness or factors relating to social life. However, physical-function items experienced as missing included sexual function, individualized items (exemplified below under *Static items*) or items that captured nuances of higher function. A person with low disease activity said:

You’ll almost be declared cured [by the HAQ] when you write there, but there is so much other stuff I cannot do….If I would walk around in [this shopping mall] for three hours, then I would have to go in here [the café] to sit and rest. Then I would be in pain….Therefore I avoid certain things. Buy in bulk? Yes, of course I can do it. But I cannot go around [all the shops in the center], for then it will be, then it is over. (M, 37, 4)

### Unclear items

Some HAQ items raised questions among participants about the intention of the HAQ developer. This items included cutting, bathing, cooking, driving cars, picking up stuff from the floor, reaching for and getting down sugar from a shelf and walking on even ground. When encountering these items, participants blamed either themselves or the instrument developer for not understanding the items. In trying to make sense of them, participants related the items to highly individual experiences or stories. For example, participants understood the ability to take a tub bath differently: It depended on whether or not they had someone helping them, they filled the bathtub first or not or the side portion of the bathtub was removable.

In general, participants made one of two kinds of interpretations of unclear items. In one case, the item would be interpreted as though it reflected a particular part of their health status:

There is a question about how to grab things from the floor, I think. And then I know that I’m always thinking, “But God, how will I answer that?”…When I have pain in that wrist, then the problem is that I have difficulty grabbing small things. That is, if a sock fell on the floor or so. It’s not that I have difficulty bending down and picking up something from the floor…[but] I find it difficult to get ahold of that little thing that might lie on the floor. That’s really what I would like to tell you, not that I find it difficult to pick up something from the floor. (W, 51, 10)

In the other case, the item would be interpreted as though it reflected a specific type of activity:

Can you cook? Yes, depending on what kind of food. [Instead,] it’s, you can cut meat? Yes, if it’s tender meat. What kind of knife am I using? I get so mad every time I answer those. I have told [my doctor], but he says this [HAQ] is made and the entire world uses it. (W, 66, 48)

In addition, patients criticized even the alternatives. For example, participants did not know how to answer when they could do something either with a special device or with the help of someone else, since the system only allowed for one answer.

### Unnecessary items

In contrast to unclear items, respondents regarded as unnecessary and skipped HAQ items concerning situations the respondent would never encounter:

Are you able to take down a pack of sugar? It is completely irrelevant to me. Can you bathe in a tub? I don’t know, I don’t have one. It appears to be designed for a specific type of rheumatism, age-rheumatism. Not for us who are young and have a completely different situation. (W, 40, 9)

Sometimes, participants saw unnecessary items as the result of standardization (detailed in the next section). Typically, participants with low disease activity also felt as though the items were too similar. One even found the repetitive process of answering to be childish:

I might think that it’s a bit childish…but there are certainly those who are sicker than me.…But there is a bit of repetition.…For me, there will be “no,” “no,” “no” all the way. But I understand that others will respond differently. (W, 68, 10)

### Static items

Participants commonly experienced having the same items in every HAQ measurement as *standardization*, which gave rise to positive and negative experiences. On a positive note, static items made participants think of areas of function they did not normally consider and thereby understood that their functional level had decreased. Negative experiences arose either from the questions or from the interaction with the physician after answering the HAQ. Participants reasoned that items irrelevant to them were there to capture divergence of the population. Despite this insight, the static items caused lack of patient engagement. This in turn made participants less attentive when answering the questions. For example, a woman who was generally satisfied with her care and perceived her disease activity and functional ability as very variable said:

Honestly speaking [answering] is a bit repetitive. Had they made some different wording and questions, one would probably get more involved and read the questions….Of course, I can walk on flat ground because I have problems with my hands….A bit too general questions. (W, 27, 9)

Similarly, she would not answer general questions from her physician candidly. For example, she would tell the physician that she was fine even when she was not and acknowledged it only when her physician saw through it. Another participant suggested that static items should be personalized and go beyond functional ability:

It is the same questions all the time. It’s a shame. It should vary. Do you cook? How is cooking? Are you eating? How long can you walk today? Have you been in town? Have you met acquaintances? Do you read? What are your interests? Because I mean, I have my son so I do not sit around on my own. But people who are alone cannot get out on their own. (W, 57; 2.5)

By contrast, if the results were supplemented with patient-physician communication after entering the data into the registry, static items were not necessarily a problem:

I do not feel like the registry standardizes care. I mean, it’s for me, it’s my values we’re talking about. I do not have the same numbers as [other patients]. So that’s good. If they fill out the same field as I do, I don’t care. Even if I have symptoms that do not fit in the registry, the physician can enter it somewhere in some system. (W, 68, 10)

### Meaning of the score

The HAQ score captured a multidimensional and continuous view of health status but was limited by the temporality and interpretation of the summative score. Understanding of the results was deepened by relating the HAQ score to other numbers or having earlier experience with the measurements.

### Capturing overall function

The summative score could represent overall function when results were compared longitudinally. The participant criticizing the item “pick up clothing” (Section “unclear items”) also acknowledged the importance of keeping the item to represent overall function:

I think it might [still] be good to ask for [the item], to get an overall picture. Because that’s why you are doing it, to see a continuous [HAQ score] or get a picture of how something has changed or not. (W, 51, 10)

However, more commonly, participants’ idea of *overall function* was either represented by a holistic assessment of item responses, or a holistic assessment of function without considering specific items. When thinking about overall function, participants related the items to a much larger story. They understood the HAQ items as a way of bringing the intricacies together into a complete picture:

I think [the HAQ] is relevant because it is good to know. Can this person get down [something that is high up], because it’s pretty elementary. I had a mother who always put things [high] up because she felt it was exquisite exercise. So I usually take after her. But some things I have abandoned: no iron, no pans of iron and things like that because it is so much work….So therefore, Teflon. But on the other hand, the iron pots are so good to cook in, and it is so infrequently that one can use them. But right then, you adapt. But on the other hand, [the questions] are important. Because [putting the items together] you can get a complete picture, if you assess the items, when seeing the patient. (W, 56, 32)

Sometimes a holistic assessment of function influenced how individual items were answered and results interpreted. A woman diagnosed 2 years ago and now working as a research nurse described how she would fill out the HAQ without thinking it through whenever she felt her disease activity had been low since her last HAQ response. By contrast, another woman called the summative score “wrong” when it did not agree with her overall picture of her health status:

It’s also difficult because we all have different diagnoses, and it [the HAQ] is supposed to pinpoint all of them. I get that, sort of. But for me, sometimes they are really stupid, sort of. So it [the HAQ score] will even give the wrong result. (W, 49, 29)

### Reflecting a temporary state

Participants noted that the HAQ only captured a temporary health state and thus gave a snapshot rather than a longitudinal understanding. This made them wish for either rephrasing the questions or higher measurement frequency. A person with generally low disease activity described:

You take the previous week [into account]. I do not meet my doctor for a year, so then I would like to [answer] maybe 3 months ago. Then I could not get my arms up here, but I can today, get those up there. So when I register today, it is great. But why was it like this 3 months ago? Why was it like that 6 months ago? Somehow [it would be good if] one registered all the time in between, before going to the doctor. So you can see that there have been some periods. A small diary or something, because it’s so easy to forget. (W, 61, 19)

Participants also viewed measurements spaced through time as a way to assess regularly the same aspects of one’s health, which gave a sense of continuity of care.

### Requiring strategies for interpretation

The summative number of the HAQ score did not instantly make sense. Participants therefore struggled with the meaning of the score relative to their general feeling of function, as mentioned above under *Capturing overall function*. They also struggled with it relative to other measurements:

There are instructions. But they do not explain really what the system is for. Some are probably wondering, “Why do I enter this?”-The obvious benefit to patients. What does the figure show? 2.94-what does it mean? There are better systems in other diseases that relate an index to a relevant measure of disease progression. (M, 57, 35)

Participants noted that it is easy to feel like they under- or over-scored in a self-assessment, for example, if they were not feeling well but were fully functional in terms of the questions contained in the HAQ. This became evident as participants discussed the results with their doctors. In particular, it was obvious for persons with low disease activity but high functional demands who scored low in the HAQ but felt their function was insufficient for their needs. If the HAQ score correlated badly with self-perceived health, participants advocated using other measurements such as clinical assessment or laboratory tests that sometimes provided different pictures. One participant who felt the HAQ questions were not for her but for sicker patients stated:

I have no need to analyze myself. I want the doctor to do it.…I do not think it reflects how I feel. The doctor should rely more on blood tests than those [HAQ] tests. (W, 64, 1)

Another strategy to overcome the struggle of interpreting the score was to relate to how the physician would interpret the numbers. That is, participants wanted to understand how the physician would extract meaning from the HAQ score.

### Effects on care and health perceptions

Participants experienced that the HAQ affected care by changing physician behavior during the appointments and affected the perception of health through their own understanding of RA.

### Effects on physician behavior

The experienced effect of registering the HAQ was either a perceived lack of effect or various degrees of influence on physician behavior. Participants sometimes saw the HAQ as a way to save time because physicians would not need to ask questions related to function. Patients perceived this either as good for the physician’s productivity solely-with no benefit for the participant-or as beneficial for both because the saved time could be used for other activities that benefited the participant.

In the case of perceived extensive effect on physician behavior, the effect depended on the summative score and the answers to individual items. For example, if the answers showed trouble opening a jar, the physician could prescribe tools to help or understand that the participant was not able to work in demanding environments such as a repair shop. Therefore, the physician’s use of individual items could determine if a participant liked or disliked the HAQ in general. A high summative score would have corresponding effects. Similarly, the HAQ could be a tool for the participant to influence care and the physician’s behavior. This even went as far as participants wanting to change the HAQ because the current items lacked an effect on physician behavior in some respect:

The questions are so fixed, and I might have other things. I have, for example, a damn hard time to lift one of my legs into the car. I need to use my hands and I would like to highlight that because I am lobbying a lot for an operation. I have so much pain that I cannot walk in certain periods. But they say that they don’t operate on young [people]. (W, 40, 9)

In addition, participants worried the HAQ self-assessment could negatively affect the quality of the general assessment because either it made doctors less prone to assessing function themselves or hard for patients to communicate their health status through the questions. As one participant observed in the waiting room:

There was some older woman there and she had a little difficulty to try to respond to that [the HAQ], so she got help from a nurse. I can, well, I think it is a pity; they might need more personal contact to get it explained and to tell more about how it is. (M, 46, 10)

### Effects on understanding of RA

While the summative score triggered questions as described in *Requiring strategies for interpretation*, the process of using the HAQ also affected participants’ understanding of RA. Patient engagement with the HAQ varied widely: Some answered as quickly as possible whereas others saw answering as an opportunity to reflect upon their functional status. Participants who did not discuss the results with the physician felt as though responding to the HAQ wasted time or the opposite—that there was no need to discuss it. Participants with the latter view instead thought the questions were valuable for gaining awareness of their problem or for documentation purposes. A participant habituated to constant pain and who, despite having symptoms considered her disease activity low, verbalized this experience:

[The HAQ can] be good for me, “Devils, I cannot do that anymore,”…sometimes you get so used to your limitations that you do not think about that or you adapt, maybe I should say. You put the stuff further down, right? So they may not be stupid questions, [rather they] get yourself to wake up. (W, 56, 32)

In contrast, the items also illustrated examples of how sick one could get, and some participants viewed that awareness as negative. This was captured by a person with low disease activity:

It also feels as if the questions do not apply to me. I mean, I feel so good after all. I can lift a bag of flour. I can walk on level ground. It just makes me afraid because that’s where I *will* be. I feel sicker. It almost feels as if I should mark that something is wrong. (W, 64, 1)

## Discussion

The findings illustrate that persons with RA mainly experience the HAQ as flawed but, despite this, as positively affecting understanding of RA and the care received. The items cause frustration, but understanding RA facilitates interpretation of one’s health. This was captured in three themes: *Problems with individual items*, illustrating HAQ limitations experienced in terms of individual items; *Meaning of the score*, experiencing HAQ strengths and limitations in terms of the summative score; and *Effects on care and health perceptions*, the positive and negative experiences of how HAQ use affects care delivery and understanding of health for persons living with RA.

The findings show how regular use of HAQ not only describes function, but also can affect care by facilitating communication between patient and physician. However, more interestingly, these findings suggest that patient conception of functional ability differs markedly from what the HAQ measures. For example, the participants experienced that the HAQ does not capture nuances of low disability (i.e., a perceived floor effect). Capturing such nuances was of great importance to participants with low disease activity but might be less obvious to instrument developers for whom such nuances are lost in comparison to patients with substantial functional impairment. That dissimilarity implies that current versions of the HAQ (in use in clinical practice) fail to generate data about functions persons with RA consider highly significant. This inadequacy could partly be traced to limited patient involvement in the HAQ development process: Patients were included only at the end of the process. Rather, the development of the HAQ departed from a professional research interest, with no consideration given to the effects on physician behaviors when the HAQ is regularly used. In addition, developers were arguably influenced by the expected outcome (in 1979) being different from the one seen today with early efficient treatment and tight monitoring.

### The literature provides a similar picture of HAQ deficiencies

Earlier research extensively used qualitative methods to study the functional needs of persons with RA (e.g., [25, 26]). A few studies have, in addition, related those findings to the HAQ, such as highlighting how context affects HAQ relevance [11], as discussed in *Unnecessary items*; noting the floor effect [12] as presented in *Missing items*; or seeing the HAQ as a mere representation of more complex activities [13], as shown in *Capturing overall function* The last of the above studies [13] made the point that tasks detailed in individual HAQ items sometimes are not themselves important but rather pose the challenge of working around them. For example, to run errands and shop is not the person’s end goal but one of many ways in which that person can get groceries home for cooking [13]. Other subthemes in the present study were not previously described.

This study shows that persons with RA have different priorities among the HAQ items and functions. In the same manner, quantitative studies have investigated patient priorities among the HAQ items and demonstrated that priorities differed [9] with rather low agreement between persons with RA (weighted κ = 0.241) [8]. Interestingly, no HAQ item seems to be unimportant to all persons with RA [15]. If the instrument were individualized by selecting the five most important items or weighted by adding additional questions about importance, then construct validity could be preserved [9]. Persons with RA freely named activities they considered important before being exposed to the HAQ and 31% of those important activities are not in the HAQ. Most of the named actions were leisure activities such as playing the guitar [8]. Leisure activities were also seen in *Missing items* of the present study.

### Conflicts between scientific value and patient value

Measuring function with the well-spread HAQ can be defended in that it enables international and historical comparisons necessary for quantitative scientific evaluations of RA interventions. However, achieving such comparability comes with the risk of poorly correlating true functional ability with the measurement (i.e., HAQ validity). For example, participants sometimes skipped the question concerning the tub bath because they did not have a bathtub. For persons who can wash their bodies and use the toilet but might be unable to take a tub bath, having or not having a bathtub would affect the score. (Because these activities are scored together, see *Hygiene* in Table 1). This specific case could be tackled by explaining to respondents that they should imagine a response rather than skip such questions.

In general, societal development ensures a constant change of human behavior and activities. Therefore, regular revision of activity-based functional scales is necessary. Such an update of the HAQ could measure the same construct as the contemporary HAQ by closely correlating them and thus still make historical comparison possible. In a sense, this means “translating” the HAQ into a new time, in the same way it has been translated into many languages.

So how would this be done? To measure function in terms of activities, one must handle divergence. This challenge is twofold: The instrument needs to capture the divergence of both the population and the activities. Answering both challenges would make it difficult to close the gap between individualized information relevant for persons with RA and general information that can be communicated in an instrument. Despite this difficulty, it is obvious from the present study that there is room for improvement. Future studies might investigate whether the same functional demand could, for example, be captured in activities patients consider more meaningful, such as social [27] or leisure activities [28]. In this way, the importance of social and leisure activities seen in this and other RA studies would be better acknowledged.

### Limitations

As seen in the present study, the character of specific items influenced the participants’ experiences. Therefore, Ekdal et al.’s context adjustments of the HAQ [15] during translation might affect transferability of the findings. Transferring the findings is especially difficult because the rationale for translating, for example, “climb up five steps” into “climb down five steps” is not explained in their publication. However, most findings in the present study are not tied to items “adjusted for context.”

Further, this study used data collected with other research questions in mind. Although the findings themselves are more important than the research question in exploratory qualitative research, additional aspects of participants’ experiences might have been better captured with a directed research question. The authors countered this by using a large number of interviews, but such breadth cannot substitute for any lack of depth. In the same manner, cognitive interviews with participants while they responded to the HAQ would have provided their immediate experiences of the HAQ. Whereas the focus of this article was instead the participant’s long-term lived experiences and creation of meaning, additional data from cognitive interviews would likely have provided supplementary information.

Finally, the selection of participants did not include persons using the HAQ as part of a research trial. It is reasonable to believe that those persons would have different experiences because the influence of the HAQ in a trial is not as tangible as it is in care, and transferability of findings to research settings would require careful consideration.

## Conclusion

The aim of this study was to explore how persons with RA experience the use of the HAQ in RA care. The findings mainly illustrate three types of experiences: wrestling with the limitations of the individual items, searching for meaning in a partly faulty summative score and handling the effects of using the HAQ. These findings suggest there is value in using a functional scale in clinical care, but the HAQ needs either revising by patients or individualized prioritization among items. Future research should investigate the relevance of items in different contexts and, to ensure relevance to persons with RA, co-produce an alternative instrument with relevant items and routines for use.

## Abbreviations

HAQ: Stanford Health Assessment Questionnaire-Disability Index
RA: Rheumatoid Arthritis
